# Teratogenicity of depleted uranium aerosols: A review from an epidemiological perspective

**DOI:** 10.1186/1476-069X-4-17

**Published:** 2005-08-26

**Authors:** Rita Hindin, Doug Brugge, Bindu Panikkar

**Affiliations:** 1 Biostatistics and Epidemiology Concentration, University of Massachusetts School of Public Health and Health Sciences, Amherst, MA, USA 01003; 2 Department of Public Health and Family Medicine, Tufts University School of Medicine, 136 Harrison Ave., Boston, MA, USA 02111; 3 Department of Civil and Environmental Engineering, Tufts School of Engineering, 200 College Avenue, Anderson Hall, Medford, MA, USA 02155

## Abstract

**Background:**

Depleted uranium is being used increasingly often as a component of munitions in military conflicts. Military personnel, civilians and the DU munitions producers are being exposed to the DU aerosols that are generated.

**Methods:**

We reviewed toxicological data on both natural and depleted uranium. We included peer reviewed studies and gray literature on birth malformations due to natural and depleted uranium. Our approach was to assess the "weight of evidence" with respect to teratogenicity of depleted uranium.

**Results:**

Animal studies firmly support the possibility that DU is a teratogen. While the detailed pathways by which environmental DU can be internalized and reach reproductive cells are not yet fully elucidated, again, the evidence supports plausibility. To date, human epidemiological data include case examples, disease registry records, a case-control study and prospective longitudinal studies.

**Discussion:**

The two most significant challenges to establishing a causal pathway between (human) parental DU exposure and the birth of offspring with defects are: i) distinguishing the role of DU from that of exposure to other potential teratogens; ii) documentation on the individual level of extent of parental DU exposure. Studies that use biomarkers, none yet reported, can help address the latter challenge. Thoughtful triangulation of the results of multiple studies (epidemiological and other) of DU teratogenicity contributes to disentangling the roles of various potentially teratogenic parental exposures. This paper is just such an endeavor.

**Conclusion:**

In aggregate the human epidemiological evidence is consistent with increased risk of birth defects in offspring of persons exposed to DU.

## Background

Depleted uranium (DU) is a man-made, radioactive, heavy metal derived from uranium ore. Naturally occurring uranium ore (rock in which the uranium concentration is approximately 1,000 or more parts per million) is mined and processed to yield a much more concentrated substance, one that is virtually pure uranium. Natural uranium exists in three isotopic forms and contains 99.274% U^238^, 0.72% U^235^, and 0.0057% U^234 ^by weight. DU, a byproduct of uranium enrichment, has an isotopic content of 99.75% U^238^, 0.25% U^235^, and 0.005% U^234^. As part of the effort to secure fissile uranium, many thousand tons of DU have been generated.

The chemical and metallic properties of DU do not differ largely from natural uranium ore or uranium oxides. However, U^238 ^has very low specific activity and particles of U^238 ^release radiation very infrequently. DU is commonly reported to retain only 60% of the radioactivity of natural uranium. This takes into consideration only the alpha emissions, but the decay process of DU with its daughter isotopes or decay products, thorium -234 and protactinium -234 adds beta and gamma emissions onto the already existing alpha emissions of U^238^. Calculations show that considering these beta and gamma emissions, DU has 75% of the radioactivity found in natural uranium [1]. DU has a radioactive half-life measured in billions of years.

DU is a dense metal; its density is 1.7 times that of lead. It reacts with most non-metallic elements; it has pyrophoric properties and may spontaneously ignite at room temperature in air, oxygen and water. These unique properties make it appealing for use in many civilian and military applications. DU is used as X-ray radiation shielding in hospitals, as counter weights for rudders and flaps in commercial aircrafts, in keels of sailing yachts and as ballast in both military and non-military airplanes. DU is used by the military for the production of distinctly powerful projectiles (e.g., bullets/penetrators, missile nose cones) and also as a protective armor for tanks.

As a projectile, a DU penetrator ignites on impact under high temperature; it has a low melting point. Further, DU sharpens as it melts making it easier to pierce heavy armor. As the projectile pierces, it leaves behind its jacket dispersing DU dust into the environment during impact. The quantity of aerosol production is directly proportional to the hardness of the armor. Normally 10–35% and up to 70% of the DU is estimated to be aerosolized on impact or when DU catches fire [2]. Most of the dust particles are reported to be smaller than 5 μm in size, i.e., of a size to be inhaled or ingested by humans [3,4]. They usually remain windborne for an extended time. There is empirical documentation that DU aerosols can travel up to 26 miles and theoretical documentation that they can travel further [5]. Once deposited on the ground the aerosols settle as partially oxidized DU dust. Potential contamination of ground water is another possibility – weathering could mobilize the metal into additional media.

U^238 ^decays primarily by alpha emission. Alpha particles rapidly lose their kinetic energy and have little penetrating power. In the decay process beta and gamma particles are emitted which are more penetrating. Alpha radiation is only hazardous when internalized in the body, but once deposited in living tissue it releases its energy in a concentrated area causing greater damage than beta or gamma radiation.

Still, large quantities of DU and/or radioactive decay products and other radioactive impurities can lead to substantial external exposure. A Geiger counter measurement by a correspondent in the recent Iraq war show that radiation emitting from a DU bullet fragment registered nearly 1000 – 1900 times the normal background radiation level. A three-foot long DU fragment from a 12 mm tank shell registered radiation 1300 times the background level. A DU tank found by the U.S Army radiological team emitted 260 – 270 millirads of radiation per hour compared to the safety limit of 100 millirads per year. A pile of jet-black dust registered a count of 9839 emissions in one minute, a level more than 300 times the average background level [6].

In the United States there are over 50 sites that have been/are engaged in developing, producing, and testing DU munitions [7]. As of 2002 it had been established that a number of other nations, including Britain, also use, produce and/or sell DU-based munitions [8]. The U.S. military deployed DU munitions for the first time during the 1991 Gulf War. DU weapons were also used in the 1994–5 war in Bosnia, the 1999 war in Kosovo, the 2002 U.S. invasion of Afghanistan and in Iraq in 2003.

Trends toward increased use of DU by industry and, more recently, in warfare suggest that there are large and growing numbers of exposed people worldwide, both at production sites and in areas where DU weapons are deployed. While there is no clear basis for estimating the number of people who have been breathing and ingesting food and water in areas contaminated with aerosolized DU particles, the ever-expanding exposure of humans and the environment to DU particles, several micrometer and smaller, mobile and inhalable, necessitates a sense of urgency to better understand this hazard.

In sum, DU bullets are made of almost pure U-238 and DU bullets and projectiles produce largely insoluble ceramic aerosols upon impact. These aerosols, largely respirable, may be a source of toxicity for those exposed. Our specific concern here is whether or not such exposure results in teratogenic outcomes. We present, however, some analysis of the toxicity of natural and non-aerosolized uranium, because the teratogenicty of soluble, natural uranium supports the plausibility of DU being a teratogen, provided it can reach the reproductive organs.

### Exposure pathways

DU can enter the body as uranium metal and as uranium oxides from the oxidized DU formed after impact with hard targets and fires. Inhalation of aerosols, ingestion and exposure through contaminated wounds or embedded fragments are all pathways of internal exposure but inhalation is the main route of human exposure both in combat and non-combat situations. Once inhaled, DU particles <5 μm can lodge deep in the lung in alveoli and can be transported by macrophages to the lymph tissues. Thereupon, live tissue immediately adjacent to (or exposed to these) imbedded particles experience infrequent but high LET alpha irradiation along with the potential for chemical toxicity. Because the micro-particles of DU are much larger than individual solubilized molecules, they can create "hot spots" of localized alpha radiation. For this to be relevant to teratogenicty, however, the particles, rather than their dissolution products, would have to reach the reproductive tissue, a phenomenon for which we are unaware of supporting evidence. (Having said this, we hasten to add that the pathways by which radiation exerts its deleterious effects on living beings are by no means fully elucidated. While discussion is beyond the scope of this paper, the "second event" theory [72], and the "bystander effect" exhibited by low level radiation [73,74] are, newly, the basis for serious re-consideration of certain propositions previously much more widely held as true.)

DU is largely non-soluble in ceramic form and is essentially comprised of three primary oxide minerals, UO_2_, U_3_O_8_, and UO_3_. UO_3 _is moderately soluble and is converted into soluble uranyl ions, UO_2_^++ ^through a stepwise reduction. The rate of oxide dissolution will vary depending on crystal structure as well as particle surface area [9]. Uranyl compounds exhibit variable solubility at different pH values, exhibiting lower solubility at circum-neutral pH and greater solubility under acidic conditions (below pH 5) [10]. A study of the dissolution properties of DU under circumstances simulating its inclusion in internal human lung secretions, demonstrated a shortest possible dissolution half-life of slightly less than 4 years [11].

To date studies of military personnel exposed to DU munitions in the 1991 Gulf War have documented the "isotopic signature" of DU in the urine of personnel 8–9 years subsequent to exposure by inhalation [12] and 7 years after exposure among those with embedded fragments [13]. These findings confirm ongoing bioavailability of soluble uranyl ions which, as the dissolution product of internal DU particles, migrate from the initial point of entry into the human body and are eventually, at least partially, excreted in urine.

The health effects related to internal exposure may result from either chemical or radiological toxicity. Solubility determines the kind of toxicity exerted by uranium. The soluble forms of uranium are more associated with toxic chemical effects while insoluble forms are associated with radiological effects. Soluble chemical forms are absorbed within days while insoluble forms generally takes months to years to be absorbed [2]. DU is organotropic and has long-term retention in its target organs, to wit the kidney and the skeletal tissue. The biological retention capability of DU in bones enhances the particulate radiation to the target organs. Though the mechanism of action of DU oxides are not clear, biodistribution studies detail DU accumulation in the bone, kidney, reproductive system, brain and lung with verified nephrotoxic, genotoxic, mutagenic and carcinogenic properties, as well as reproductive and teratogenic alterations [14]. However the subject of inquiry from the vantage of teratogenicity is the potential for DU exposure of egg and embryo and of sperm and cells involved in spermatogenesis. (New information on how radiation affects cells, as noted in the first paragraph of this section, may contribute to elucidation of how reproductive tissue could be damaged indirectly.)

### Theoretical Basis for Human Teratogenicity

With a few exceptions, it is only since the late 1980s that uranium's reproductive toxicity has been studied using animal models. Most of the past 15 years of published research on the topic comes from two groups, Domingo, and others working at the University of Barcelona in Spain and McClain, Benson, Miller, Pellmar and others affiliated with the Armed Forces Radiobiology Research Institute (AFRRI) in the United States [15-17]. With at least 6 published studies on topic, Domingo et al. have demonstrated that both oral and subcutaneous administration of UO_2_^++ ^to female mice engender decreased fertility, embryonic and fetal toxicity including reduced growth and malformations (cleft palate and skeletal defects) and developmental ossification variations. From their maternal animal exposure studies the members of Domingo's group concluded that it was chemical toxicity, not radiation that resulted in teratogenicity [15,18-22]. The chemical reproductive toxicity of DU could act at the molecular level (damaging DNA and RNA), at the cellular level, and/or at the organ level, affecting organs including the testes, placenta, and embryo/fetus.

Two studies of orally dosed male rats that were conducted decades earlier demonstrated substantial degeneration of testes and impact on germ cells; another more recent study provided some similar evidence [21,23,24]. Very recent research suggests that uranium mimics estrogen in mice [25].

The AFRRI studies were funded in 1994 by the military in order to investigate the toxicity of embedded DU fragments [26]. These studies, using a rat model, have demonstrated that DU pellets embedded in male rats led to elevated uranium concentrations in the testes, and that pellets embedded in females led to detectable uranium in the placenta and to "very low levels" of its accumulation in the fetus, though there was no "overt" teratology. There is preliminary evidence of delayed reproductive impact of embedded DU among female rats; the probability of decreased litter size increased in proportion to time since embedding. Several rat studies by the AFRRI group have shown that embedded DU pellets are mutagenic [16,26,27]. In their human studies, McDiarmid et al. found "subtle perturbations" in indices of reproductive health among their shrapnel-exposed human subjects [28].

A Chinese study of reproductive toxicity of enriched uranium noted damage to genetic material, dominant lethality and skeletal abnormalities in fetal rats. Chromosome aberrations in spermatogonia, DNA alterations in spermatocytes and strand breakage in sperm were specifically notified [29]. *In vitro *experiments documented extensive DNA damage when UO_2_^++ ^was added to DNA in the presence of an electron donor. Since DNA is particularly dense in sperm-forming cells, such cells may be especially susceptible to UO_2_^++^-derived damage. In sum, aerosolized DU is a vehicle for internal delivery of a DNA-tropic substance that is both a heavy metal and an alpha particle emitter.

Chromosomal instabilities have also been documented in humans. In 1997 Zaire et al reported finding increased frequency of sister chromatid exchanges in cells of uranium miners [30]. And in 2001 McDiarmid reported a similar finding among 1991 Gulf War veterans with embedded DU shrapnel [28]. In 2003 Schroder et al. documented chromosomal instability (in the form of increased frequency of dicentric and centric ring chromosomes) among sixteen 1991 Gulf and Balkan war veterans who believe they were exposed to DU via dust inhalation [31]. Mutagenicity of uranium was also observed in residents living near uranium mines. Au et al. looked at non-smokers who resided near uranium mining or milling sites in Texas, but had not worked in the uranium industry [32,33]. They found that residents living near the uranium mining and milling sites had higher frequencies than controls of aberrant cells, chromosome deletions and chromosomal aberrations.

Consideration of evidence regarding the teratogenicity of heavy metals other than uranium is also relevant for estimation of the hazard that DU poses. First, elegant studies of other heavy metals suggest study designs that may be emulated; second, observed associations can inform and contribute to the choice of outcomes for DU studies since various heavy metals may have similar modes of action. Accumulated knowledge of heavy metal teratology is quite extensive; animal teratogenesis by a variety of heavy metals, and human teratogenesis by, at minimum, lead and mercury, are long established [34]. A 1996 study of human anencephaly vis a vis parental and *in utero *ambient exposure to lead, mercury and vanadium is exemplary. Levels of these three substances were measured in the brain, kidney, liver, and lung of 20 anencephalic and 20 control fetuses, all of which were conceived in an area of Venezuela where these heavy metals were (are?) being constantly released into the environment by "a petroleum empire [that] has grown indiscriminately". In sum, the researchers stated that " [m]ercury and Pb were significantly increased (p less than 0.001) in kidney and liver of anencephalic fetuses. Vanadium was detected exclusively at brain level, being significantly higher in controls (p less than 0.05).... In conclusion, Hg and Pb are toxic elements present in the Eastern coast's environment that should be seriously considered for cause/effect studies when the etiology of anencephaly in this region is considered..." [35].

### Epidemiological and other population studies of DU teratogenicity

Investigation of DU teratogenicity in exposed populations is constrained by the rigor with which such populations can be accurately identified [36]. Parental exposure to a possible teratogen can be established on, at least, two different levels. Especially when exposure is from the ambient environment rather than from individual activity (e.g., consumption of a particular substance), documentation at the intra-individual level, through the use of assays measuring biomarkers (as described just above ([35]) enhances clarity. There have been no studies of DU's teratogenicity with concomitant biomarker documentation of individual DU exposure. The first report that assessed, and supported, the feasibility of use of biomarkers for measurement of DU and other environmental chemical exposures during military deployment has recently been reported [37].

On the ecological level, being at-risk for exposure requires documentation of i) the presence of the suspected teratogen in the ambient environment and ii) the location of the allegedly exposed at the time(s) of (peak) exposure. With only ecological-level information regarding exposure, there is a limit to the clarity that can be achieved regarding the biological activity, in a given individual, of the ambient toxin.

Exposure to DU aerosols, most often the "by-product" of war, usually occurs along with other possible, probable and known risky exposures. This poses a particular challenge to inference: distinguishing the role of DU, per se, and requires weighing the evidence as thoughtfully as possible. Indeed, engagement with this challenge is one of the main objectives of this paper. The most important strategy to address this type of challenge is to relate findings in various settings, especially, unusual settings, in which populations received exposure. To the extent that the occurrence of possible risk factors for teratogenesis varies between contexts, the consistency of DU exposure across those contexts increases the probability that it is the (or an) explanatory factor.

### Inclusion Criteria for Review

In this article, consideration of DU's reproductive toxicity includes its mutagenic and teratogenic potential, i.e., activity as a pre-conception mutagen affecting the female or male germ line as well as conception-to-birth impact. Studies that consider paternal DU exposure are, uniformly, included. While animal studies pertaining to a variety of endpoints have been referenced above, the human epidemiological studies considered here are limited to those that focus on physical malformations. Epidemiological-type studies of congenital malformations and uranium (though not depleted uranium) exposure are included; studies of populations exposed to other heavy metals and to other sources of low-level radiation are not. (But, there is only one study of congenital malformations among residents of a uranium mining area.) The several studies of reproductive outcomes among military personnel who served in the 1991 Gulf War, though not specifically in areas of Iraq where DU munitions were employed, are included. Findings of studies of all 1991 Gulf War veterans are interpreted accordingly – in these studies, Gulf War deployment, though used as an indicator of DU exposure means only substantially increased probability of exposure compared to non-Gulf War populations.

### The Studies

#### Socorro Case Study

The earliest DU "research" is an instance of preliminary, "shoe leather" epidemiology that was carried out by community activists in the United States [38]. Socorro County, New Mexico is a sparsely populated rural area downwind of a DU-weapons testing site, the New Mexico Institute of Mining and Technology's Terminal Effects Research and Analysis (TERA) division [39]. In a letter addressed to TERA, a community activist enumerated birth defects among infants born in Socorro County between 1979 and 1986. The writer said she was referencing cases reported in the State of New Mexico's passive birth defects registry, i.e. a system that aggregates reports of birth defects though it does not have staff who seek out cases. The writer also reported two infants with birth defects in 1985 that were known to her but not recorded in the registry. In a county with about 250 births/year the writer reported 5 infants born with hydrocephalus (though 1 of 5, she stated, was not recorded in the registry). There was nothing remarkable about the 16 other abnormalities she enumerated.

All the instances of hydrocephalus occurred between 1984 and 1986. In 1998 another community activist requested and received a count of all hydrocephalic births in New Mexico and in Socorro County for the years 1984 – 1988 from the State Department of Health. The registry report documented a total of 19 infants born with hydrocephalus in New Mexico during those 5 years; 3 of them were Socorro County residents. Socorro is less than 1% of the State's population. Though there are several reasons why these findings cannot be considered the results of a methodologically rigorous investigation, the data are provocative, are a cause for concern and should be followed up. It would also be most valuable to seek out and aggregate other "shoe leather" findings.

#### ABDC Case Series

Hydrocephalus is also a component of the congenital malformation syndrome Goldenhar Syndrome. The Association of Birth Defects Children (ABDC) identified Goldenhar's as apparently occurring in excess among the offspring of male 1991 Gulf War veterans [40]. ABDC generates a birth defects registry by solicitation of reports of children with birth defects from parents. With thousands of parents reporting their children's defects and possible risk factors, including 1991 Gulf War exposures, the data brought the apparent excess to light. Goldenhar Syndrome is a variable cluster of eye, ear, face and vertebral malformations, often includes hydrocephalus.

#### Araneta et al/Goldenhar Cohort Study

As an attempt to provide a prompt follow-up to the ABDC "alarm", Araneta et al. utilized military hospital records to compare prevalence of Goldenhar Syndrome among offspring of 1991 Gulf War veterans and offspring of a cohort of non-deployed veterans [41]. Though the recorded prevalence of Goldenhar Syndrome was three-fold higher among the 34,000+ offspring of deployed veterans (14.7/100,000) than among the 41,000+ offspring of non-deployed veterans (4.8/100,000), the differential, based on 5 and 2 cases, respectively, of the rare syndrome, was not statistically significant. The utilization of military hospital births is an additional challenge to detection of association. Only active duty military personnel and their wives deliver at military hospitals. It is possible that one or more debilitating wartime exposures led both to termination of active duty status and increased risk of siring an affected child; if so, the choice of study population leads to bias against observing an association. From an epidemiological perspective, this study was designed to test a particular hypothesis and, though sample size obviated statistical association, it affirmed a strong likelihood of increased risk.

#### Dr. Gunther's Reports of Field Observations

Shortly after the 1991 Gulf War a German physician working in Iraq began to publicize his observations of catastrophic and ongoing ill health and distinctive patterns of abnormality among the Iraqi population. Gunther, the president of the Austrian-based humanitarian and relief organization Yellow Cross International had spent much time in Iraq and has had an appointment as Professor of Infectious Diseases and Epidemiology at the University of Baghdad. Though he has not published systematic data, he is convinced by his clinical experience that DU munitions are the cause of much of the horrific human toll that he saw. As early as 1996 he published a book entitled "Uranium Projectiles: Severely Maimed Soldiers, Deformed Babies, Dying Children" in which he presented his impressions of post 1991 Gulf War ill health, both in words and photographs. A second, tri-lingual (German, English, French) edition was published in 2000 and again the volume is more images than text [42]. Of 28 photographs of diseased or malformed children in the 2000 edition, four pertain to the classical infectious diseases associated with poverty and poor hygiene and two to malnutrition; the captions of the photographs of four malformed children indicate hydrocephalus.

#### Basra, Iraq Registry Studies

There are reports on the internet of papers delivered at international conferences, held in Iraq, on DU. The reports describe work being carried out by a clinical epidemiology research team in one of the three major maternity hospitals in Basra, Iraq [43,44]. Basra, a city of some 1.6 million, is the second largest Iraqi city and is in the region that was heavily exposed to bombardment with DU munitions during the 1991 Gulf War. Since 1989 the Basra team has kept a congenital malformations registry; each newborn is assessed before discharge from hospital [45]. Both internet-published reports references data for the 1990 birth cohort as the non-exposed group. There were between 9,845 and 13,905 births included in the registry annually between 1990 and 2000. At the World Uranium Weapons Conference held in Hamburg, Germany October 16–19, 2003 a member of the Basra team reported data for total congenital malformations in 2001. Table 1 gives the number and rate of total malformations recorded in the registry by year.

**Table 1:** Congenital Malformations Surveillance Data from One of Basra's Three Main Maternal and Children's Hospitals, Iraq 1990 – 2001 [43, 44] (Number and Rate of All Malformations Combined)

Year	No. of Births	No. of congenital malformations	Congenital malformation
			
			incidence rate/1000 births
1990	12,161	37	3.04
1991	9,845	28	2.84
1992	11,800	23	1.95
1993	12,416	28	1.31
1994	12,250	36	2.93
1995	10,576	46	4.35
1996	10,470	48	4.56
1997	13,653	32	2.34
1998	10,186	79	7.76
1999	13,905	136	9.78
2000	12,560	221	17.6
2001	11,445	254	22.19

Initially reporting on only the 1990–1998 data, the authors observed "an apparent increase in the incidence rate from 1995 upwards". Therefore, " [t]o improve statistical efficiency of the data collected and overcome small numbers of cases recorded, the pattern and incidence of congenital malformations are grouped into two periods, 1991 to 1994 and 1995 to 1998."

Incidence data, by class of malformation were reported for the time periods 1990, 1991–1994, 1995–1998 and 1999–2000; the data are redacted and presented in Table 2. (In particular, rates have been calculated.) Category names and data for all malformation categories are presented below exactly as they were specified in the internet articles. Malformation categories in which cases were first documented in 1999–2000 are listed only in the Fasy report, which includes data through that later time point. Of particular note, the report of data through 1998 does not include hydrocephalus and the report of data through 2000 indicates no diagnosed cases of hydrocephalus between 1990 and 1998 at the study hospital. However, by personal communication one of the Basra researchers confirmed to the authors that there were infants diagnosed with hydrocephalus born during the years 1990 – 1998, indeed that hydrocephalus was more commonly diagnosed during the latter part of the 90's than previously [45]. No information beyond that given in the table is provided regarding the particular cases included in each grouping.

**Table 2:** Redacted Presentation of Congenital Malformations Surveillance Data: All Births at One of Basra's Three Main Maternal and Children's Hospitals, Iraq 1990 – 2000 (Malformation Categories Exactly as Given by Basra Clinicians) [43, 44]

Year(s)	1990	1991–94	1995–98	1999–2000
Total number of births	12,161	46,311	44,885	26,465
Class of malformation (Rate per 1000 births (No.))				
All malformations combined	3.04 (37)	2.48 (115)	4.57 (205)	13.49 (357)
***Cardiovascular***				
Congenital heart diseases	.16 (2)	.39 (18)	.96 (43)	1.36 (36)
***Central Nervous System***				
Anencephaly	.25 (3)	.30 (14)	.36 (16)	1.74 (46)
Hydrocephalus	*see text*	*see text*	*see text*	1.47 (39)
Meningomyelocele	.70 (9)	.43 (20)	.94 (42)	1.13 (30)
***Musculoskeletal***				
Achondroplasia	.25 (3)	.06 (3)	.20 (9)	-- (4)
Arthrogryposis	0	0	0	-- (1)
Phocomelia	0	--(1)	.11 (5)	1.21 (32)
***Multiple Congenital Malformations***				
Multiple Congenital Malformations	.58 (7)	.65 (30)	1.09 (49)	4.23 (112)
***Orofacial***				
Cleft lip & palate	-- (1)	.15 (7)	.20 (9)	.79 (21)
***Congenital***				
*Chromosomal aberrations*	.16 (2)	.13 (6)	.33 (15)	.30 (8)
***Gastrointestinal***				
Oesophageal atresia	-- (1)	-- (3)	-- (3)	.42 (11)
Omphalocele	-- (2)	.11 (5)	.11 (5)	0
Imperforate anus	-- (1)	-- (1)	0	.19 (5)
Diaphragmatic hernia	-- (4)	-- (1)	-- (2)	-- (3)
***Genitourinary***				
Bladder extrophy	-- (2)	-- (1)	-- (2)	-- (1)
***Other***				
Cyclopia	0	0	0	-- (2)
Icthyosis	0	.11 (5)	.11 (5)	.23 (6)

#### Diwaniah, Iraq Registry

Research by Al-Shammosy pertains exclusively to neural tube defects and describes the findings of a study that identified all year 2000 births with a neural tube defect (NTD) at the Diwaniah maternal and children's hospital [46]. Diwaniah is a city slightly north and east of Basra and, like Basra, is in the area heavily bombarded with DU munitions during the 1991 Gulf War. The overall 2000 NTD incidence rate in Diwaniah was 8.4/1,000 (73 cases of NTDs among 8,707 births). Table 3 gives the 2000 incidence rates for particular NTDs. Al-Shammosy raises concern about universal maternal exposure to DU while stating, without providing any supportive data, that he investigated other possible risk factors for NTDs but found nothing unusual. Al-Shammosy also cited a previous report on NTDs among Diwaniah newborns, 7/98–2/99. During that 8-month period there were 33 cases of NTDs among 6124 births, 5.4/1000.

**Table 3:** Prevalence and Type of Neural Tube Defects among Newborns: Diwaniah Maternal and Children's Hospital, Iraq 2000 [46]

Type of Neural Tube Defect	Year 2000 Rate/1000 (N)
Anencephaly	3.1 (27)
Meningocele	2.4 (21)
Meningomyelocele	2.3 (20)
Encephalocele	0.6 (5)

#### Iraqi Congenital Abnormalities Clinic

This clinic-based study of congenital abnormalities among Iraqi stillborns and children <= 2 years of age,[47] provides the number of children or stillborns with specific abnormalities seen among 1038 cases in 1989–90 and among 945 cases in 1992–93 (pre vs. post war). The percent of patients seen whose malformation was anencephaly or hydrocephalus rose from 0.5% to 1.1% and the percent of patients seen with skeletal abnormalities rose from 2.8% to 4.6%. The abstract does not give any information on parents' residence or fathers' occupation.

#### Mostar Cohorts

Sumanovic-Glamuzina et al. investigated the prevalence and type of congenital malformations among infants born in the West Mostar region of Bosnia and Herzegovina during the years 1995 and 2000 because of concern that DU munitions were used during the 1991–1995 war in that region [48]. Live and stillborns were examined 0 – 3 days postpartum, though a few malformations were noted later. This study is so sorely compromised methodologically that it defies interpretation. For example, the protocol for detecting birth defects was different for the two cohorts. Also, the distinctions between the two-birth cohorts vis a vis exposure is unclear.

#### US Veterans Cohort Study

There are no studies of birth defects among offspring of American or allied veterans of the 1991 Gulf War which distinguish veterans by the area of Iraq in which they were stationed. Thus, all 1991 Gulf War veterans' studies, from the point of view of assessing the teratogenic capacity of DU munitions, have a "contaminated" exposed group. The war-exposed group is comprised of individuals whose DU exposure would have ranged on a continuum from none to heavy, and they cannot be sorted further.

The 2000 and 2003 studies by Araneta et al of birth defects among offspring born to 1991 Gulf War veterans have their study populations and comparison criteria rigorously defined [49,50]. In the larger, updated study data from all areas in the United States in which there were active birth defects surveillance systems operating during 1989 – 1993 were analyzed. Occurrences of individual birth defects (aggregated into 48 groupings) among offspring of male and female 1991 Gulf War veterans and 1991 non-deployed veterans were ascertained through the first year of life. Those data provide the basis for comparisons regarding birth defects among offspring of gender-specific groups of veterans, including deployed veterans conceiving children pre-war vs. post-war and veterans conceiving children post-war, deployed vs. non-deployed. Analysis relied on statistical tests to identify significantly different prevalence rates for individual groupings of birth defects among cohorts being compared. An infant with multiple anomalies was usually included in several birth defect groups. There were 308 infants conceived post-war to deployed female veterans and 4,648 infants conceived post-war to deployed male veterans. The statistically significant findings are detailed in Table 4.

**Table 4:** Statistically Significant Findings in the Study by Araneta et al. (2003) [50] of Birth Defects among US Veterans Deployed and Not Deployed in the 1991 Gulf War

Birth defect	Comparison groups	Relative risk (95% c. i., p-value)
*Father was a veteran*		
Aortic valve stenosis	Infants conceived post-war, to deployed fathers vs. non-deployed fathers	6.0 (1.2–31.0, p = 0.026)
	Infants of deployed fathers, those conceived post vs. those conceived pre-war	^16.3 (0.09–294, p = 0.011)
Renal agenesis or hypoplasia	Infants of deployed fathers, those conceived post vs. those conceived pre-war	^16.3 (0.09–294, p = 0.011)
Tricuspid valve insufficiency	Infants conceived post-war, to deployed fathers vs. non-deployed fathers	2.7 (1.1–6.6, p = 0.039)
*Mother was a veteran father may or may not have been a veteran*		
Hypospadias and epispadias	Infants conceived post-war, to deployed fathers vs. non-deployed mothers	6.3 (1.5–26.3, p = 0.015)

^5 of 4,648 offspring conceived post-war were so affected vs. 0 of 6,863 conceived pre-war; logit estimator method used for statistical significance testing.

#### Study of Births to US Active Duty Military

Two earlier records based studies found no association between the (from our perspective) over-expansive exposure criterion (Yes/No deployment in the 1991 Gulf War) and occurrence of birth defects in offspring born post-War to military personnel. Unfortunately, due to design features neither study can contribute to elucidating the nature of the relationship between parental DU exposure and congenital malformations in offspring. Cowan et al. took advantage of the maternity option available to active-duty military personnel and their wives and used military hospital birth records from 1991 – 1993 to assess the occurrence of congenital malformations among offspring of veterans subsequent to deployment in the 1991 Gulf War or elsewhere [51]. Study offspring of male Gulf War veterans numbered over 30,000 and of females Gulf War veterans nearly 4,000. Limiting post-war assessment of (reproductive outcomes among) wartime military personnel to those remaining on active-duty excludes individuals for whom war-induced debility engenders early termination of service. It is surely possible that on the population level wartime DU exposure related to termination of service; but if this is the case, the exposed cohort is also depleted of those who are the hypothesized at-risk population. Cowan et al. found no statistical association between deployment status or length of deployment and all birth defects, birth defects considered frequent and severe enough to pose a public health problem, or several specific categories of those birth defects considered more severe. What remains unknown is the extent to which this finding of no association is related to the a priori exclusion, from the pool of potential study parents, of persons who'd left service.

#### Study of US Military Offspring, Self-report

There is one other large cohort study that compared reproductive outcomes among 1991 Gulf War veterans and non-Gulf veterans. From an epidemiological perspective, a distinctive feature of the study by Kang et al. is that data on pregnancy outcomes were acquired by self-report and thus are susceptible to recall bias [52]. Beginning in 1996 and using stratified random sampling, Kang et al. assembled two gender and unit (active/reserve/National Guard) diverse study cohorts, each comprised of 15,000 veterans. Seventy-five percent of Gulf War veterans and 65% of non-Gulf veterans completed a study questionnaire or phone interview that provided information about birth defects (and other births outcomes) among the offspring of their first post-deployment pregnancies. There were 3397 offspring of male and female Gulf War veterans and 2646 of non-Gulf War veterans. Prevalence of any birth defect, to re-iterate, by self-report, was elevated among offspring of Gulf War veterans of both genders. The prevalence remained elevated after statistical adjustment for various potential confounders as well as after exclusion of about 1/3 of all reports of defects because reviewers, blind to parents' deployment site, considered the actual (self) report suspect. The authors' classification of the 206 remaining defects was only minimally informative: Data analysis did not separate offspring of exposed fathers and exposed mothers; nor were the sub-categories particularly informative. Among the 3397 offspring of the combined male and female Gulf War veterans there were 111 "isolated anomalies", and 35 other defects grouped in 6 categories, and there were 40 "isolated anomalies" and 20 other defects among the 2646 offspring of the non-Gulf War veterans.

#### Mississippi National Guard Study

In response to a report in the popular press that there was a cluster of birth defects and other health problems among offspring born to members of two Mississippi National Guard units deployed in the 1991 Gulf War, Penman and Tarver conducted a highly detailed and labor-intensive assessment [53]. They were able to gather data for ninety percent of the 284 members of the two units. At the time of the investigation, the 254 contacted veterans had begotten 67 offspring. There was nothing unusual, statistically or otherwise, about the 3 major and 2 minor congenital malformations observed among the offspring. However, sixty-seven is a small sample population of births to study malformations and assess statistical significance.

#### British Veterans' Offspring Study

In 2004 Doyle et al. reported the results of a mail survey about post-war reproductive outcomes among 1991 British Gulf War and other veterans [54]. The survey was sent to all UK armed services personnel stationed in the Gulf between 8/90 and 6/91 and a stratum-matched sample of other active duty armed services personnel. Data gathering occurred between 1998 and 2001. The study was designed to gather information on infertility, miscarriage and birth defects, the latter among fetal deaths as well as live births, and to obtain clinical verification of self-reports whenever possible.

24,379 or 53% of the surveyed male1991 Gulf War veterans and 18,439 or 42% of the male veterans deployed elsewhere responded to the survey. Response rates among the 1200+ female personnel in Gulf War-deployed and elsewhere-deployed cohorts were somewhat higher. Because of disappointingly low response rates, an intensive tracing study was undertaken to try to assess whether non-response introduced irremediable bias; its results were not damning. Data were presented that indicated that about half of reported events could be confirmed clinically [55].

Malformations were grouped into 11 classes and 18 sub-classes according to the European Registry of Congenital Anomalies (EUROCAT) and, as well, into two additional classes, malformations of tissues derivative from the embryonic cranial-neural crest and metabolic/single gene defects. The incidence of several classes and sub-classes of malformations were more common among the offspring of Gulf War deployed veterans vs. non-deployed veterans, when the database included all self-reported malformations. Restricting analysis to the 55% of the malformed infants whose abnormalities were clinically verified yielded not only wider confidence intervals around the relative risks (as occurs because of smaller sample sizes) but also "a general shift of the point estimates towards the null" (p. 80). In this latter analysis there were no excesses of malformed infants that were statistically significant.

#### Canadian 1991 Gulf War Veterans' Health Study

The Goss-Gilroy Inc. consulting firm was hired by the Canadian Department of National Defense to study the health (including reproductive outcomes) of personnel who served in the1991 Gulf War. All 4,262 Canadian veterans of the 1991 Gulf War were mailed a questionnaire and 73% responded/enrolled. Sixty percent of non-deployed veterans selected as controls enrolled. Information about birth defects was sought for all offspring, i.e., those born before, during and after the 1991 war. The study found that, compared to non-deployed veterans, deployed veterans (self-) reported higher rates of congenital malformations among offspring born during each of those three time periods – suggesting the possibility of reporting bias. Confounding the challenge of interpreting the study findings is the fact that the information reported about particular birth defects among children born to deployed and non-deployed veterans was not stratified by time period (before, during, or after military service), rather all classes of defects were compared among all children of deployed and non-deployed veterans.

#### Australian 1991 Gulf War Veterans' Health Study

This study attempted to gather extensive data (including reproductive histories) on all Australian 1991 Gulf War veterans and on members of a comparison cohort. Just over 80% of Australian's 1876 veterans participated, a somewhat smaller percentage of controls. Reported post-War birth defects were similar among offspring of deployed and non-deployed veterans.

#### Kuwaiti Congenital Heart Disease Study

Based on clinical suspicion of increased anomalies, Abushaban et al. reviewed the annual frequency and type of congenital heart defects among all Kuwaiti newborns during the years 1986 – 1989 and 1992 – 2000, i.e., pre- and post- the 1991 Gulf War [56]. Abushaban et al. report that during the 1991 Gulf War Iraqi soldiers, before leaving Kuwait, set fire to an astounding 770 Kuwaiti oil fields and the environmental damage, the air, water and land pollution was huge. Also, *inter alia*, there was a fire at a munitions storage site that housed DU munitions.

The average annual incidence rate of congenital heart disease was 39.5/10,000 for the years 1986 – 1989 and 103.4/10,000 for the years 1992 – 2000, with a suggestion that the rate began to decline in the most recent years. For 13 of 17 sub-categories of congenital heart disease, there was a statistically significantly higher incidence rate in the latter time period.

#### Shiprock Uranium Mining Area Study

The one other epidemiological study that bears on the issue of DU and birth defects is the 1992 study by Shields et al. that assessed birth outcomes among Navajos working and/or residing in the Shiprock, New Mexico uranium mining area [57]. Utilizing a nested study design, cases and controls were identified among the 13,000+ consecutive Navajo births at the Shiprock Indian Health Service Hospital during the years 1964–1981; cases included infants born with congenital anomalies, developmental disorders, stillbirths and non-injury-related infant deaths. Most children born at Shiprock continued to receive medical care there. Record review identified 320 singletons with "defective congenital conditions"; matched controls were selected. Inclusion in the case-control study was limited to the 266 (83%) dyads where families of both cases and controls were found and interviewed. There were 5 dichotomous measures of uranium exposure: father employed in mining or milling uranium, father living within 0.5 miles of a mine, likewise mother, father living within 0.5 miles of a mine dump or tailings pile, likewise mother. Paternal occupational data measuring exposure to radon daughters and information about grandparents' uranium exposures were gathered in the few instances such information was accessible.

The review of birth charts and subsequent health service records revealed that 140 of the 13,329 (1.1%) children born in the Shiprock uranium mining area 1964 – 1981 were identified as having congenital malformations. The authors grouped the congenital malformations observed among their case infants into five sub-divisions: chromosomal disorders, single gene mutations, multi-factorial conditions with morphological anomalies (excluding hip), hip dysplasia and dislocation, and teratogenic effects and other outcomes of known causes. According to the categories of Shields et al. there were 97 cases with multifactorial conditions, 20 with hip defects and fewer than 10 with each of the other categories of birth defects. (Note that Shields et al. chose to separate out hip dysplasia and dislocation because of the known high prevalence of that congenital malformation among Navajo.) But with 5 sub-divisions of malformations and 5 types of exposures, data analysis through statistical testing revealed only non-statistically significant associations – stymied by small numbers and the confusion of multiple tests.

A limited re-analysis of the available birth defects data is offered in Table 5. Rather than separate statistical analysis of each outcome category by each type of exposure, the data provided for the two more populous defect categories were used to allow for summing (without weighing) the three types of paternal and two types of maternal exposure. For example, an individual father who worked in a mine, resided within a half mile of that mine and resided within a half mile of mine tailings would contribute 3 points worth of exposure. This analysis suggests that there could be associations in the data that were not discerned by the original analytic method and that reanalysis may be warranted.

**Table 5:** Uranium Exposure and the More Common Categories of Congenital Malformations among Navajo in the Shiprock Area, New Mexico Re-Analysis of the Data of Shields et al. 1992.

Congenital Malformation	Count of Exposures				
	
	Number of dyads	Fathers (max/individual = 3)		Mothers (max/individual = 2)	
		
		Cases	Controls	Cases	Controls
Multifactorial conditions with morphologic anomalies (excluding hip)	97	42	27	23	20
Hip dysplasia and dislocation	20	16	4	13	2

#### Other reports

There are numerous references in the news media regarding both i) an excess of birth defects and ii) the occurrence of unusual birth defects among infants born after 1991 to returning Gulf War veterans and to residents in the area of Iraq exposed to DU munitions in the war. Of greatest interest is a 1999 article in the British newspaper The Guardian "Victims of a war they never saw" [58]. The article provides quotations from interviews with several Iraqi researchers that invoke concern. Most strikingly, an Iraqi physician then working in a Basra maternity hospital with 20 – 30 deliveries daily was quoted as saying "August – we had three babies born with no head. Four had abnormally large heads. In September we had six with no heads, none with large heads and two with short limbs...." In October, one with no head four with big heads and four with deformed limbs or other types of deformities." The Western-trained physician-geneticist, author of the previously cited 1994 report on the changing pattern of birth defects observed among genetics clinic patients, pre vs. post 1991 Gulf War [47], is quoted as follows: "We're getting mothers as young as 20 giving birth to Mongol babies... My research shows that the number of children born with Down's syndrome-type defects since the war has tripled."

Possibly the most vivid and widely seen image in this country is the LIFE magazine cover photograph of the child with phocomelia born to a recently returned 1991 male Gulf War veteran [59]. There have been more recent, prominent articles in the popular press as well. Japanese peace activists have also produced a volume of searing photographs of damaged children [60]. The lay press has reported that there has been an increase of birth defects in the region in Holland where an airplane, with DU as its ballast, crashed in 1992 and in the region of Remscheid, Germany where a U.S. army plane with DU ballast crashed in 1988 [61,62].

### Framework for assessment of DU teratogenesis from an epidemiological perspective

The most compelling strands of evidence regarding the possibility that DU aerosols are teratogenic are:

i) Findings of the substantial array of DU research undertaken from the vantage of radiation physics, cell biology and animal experimentation;

ii) The documentation of birth defects in southern Iraq, including comparison data from before use of DU munitions in that area.

Documentation of elevated prevalence of the birth defect hydrocephalus among infants born downwind of an American DU munitions testing site is of particular interest in that hydrocephalus has been identified in other contexts as possibly associated with DU exposure.

There are two other sources of information that, with additional detail could be much more informative:

i) The methodologically precise study by Araneta et al. [49] which was designed to assess whether all and particular congenital malformations were more common among offspring of 1991 Gulf War veterans than among other veterans did that well. If it were possible to re-analyze those data and incorporate information on theatre of service of individual veterans, that would provide a truer proxy for DU exposure, rather than the imprecise approximation of Yes/No deployment in the war.

ii) A more data oriented presentation of the basis for his clinical impressions would be most welcome from the physician president of the Yellow Cross International.

There is also a small group of other studies that are methodologically compromised; yet they are relevant in so far as they can be considered in relation to other data. Studies that relied on parental "self-report" of birth defects are given less weight because of the potential for biased recall. Low overall response rates, adds another potential source of error for telephone or mailed surveys – the possibility that sub-groups with differential risk may have had different likelihoods of responding.

A multiplicity of considerations bear upon the establishment of causality and, especially regarding birth defects, epidemiological inference relies on input from a variety of other disciplines. The five now classic epidemiological principles of inference (time order, strength, consistency and specificity of association, and coherence) frame the assessment of teratogenicity. Kline et al. describe coherence as "an ultimate criterion, one in which the observed association is weighed against all previously existing theory and knowledge" [36]. Unusual for the early phase of epidemiological investigation of potential teratogenesis, regarding DU there already exists a body of research on the molecular, cellular and animal model level that indicates plausibility. Indeed, this body of research is substantial enough that of itself it generates concern.

Regarding DU and birth defects, the ability to assess the strength, specificity and consistency of the association, and thus the overall ability to discern causality, are impacted by the absence of i) research that documents exposure on the intra-individual level and ii) research that purposefully attempts to distinguish the role of DU from co-occurring exposures. Nor is there a scale according to which the hazard of a given amount of external exposure has been calibrated. The available high quality bioassay that quantifies an individual's internal DU exposure relies on mass spectrometry of 24-hour urine samples, at a cost of about $1,000 [63]. Efforts to theoretically model dispersal of DU aerosols [3,64] or the amount of DU present in the soil and air some months after use in war [4,65,66] are very recent.

The pattern of secondary dispersal of DU particles is another outstanding question. Yet others include: What is the nature of the relationship between amount of DU inhaled or ingested and subsequent internal DU activity? How do inhalation and ingestion exposure compare? How significant is proximity at the very time that DU is burning? Is risky proximity measured in meters or kilometers? Is the relationship between proximity and hazard linear, on what scale, or is it non-linear? Are wind patterns as important as proximity? Since DU travels by wind and is respirable and ingestible long after the DU fires are out, what risk is attached to ongoing residence in or re-locating to a region where DU munitions were used in the past?

There are also particular challenges to epidemiological investigation posed by the fact that birth defects are the outcome under study. Depending on method of categorization, the prevalence of birth defect categories can be 1 in 1,000, 1 in 5,000 or less. Even with multi-thousand cohorts, unless the actual size of the association is quite large, statistical power to detect an association in a cohort study will be compromised. The implication is that finding statistical association is informative, but finding "no statistical association", absent adequate statistical power, is not definitive. Undertaking case-control studies (which would not be affected by the rarity of the study outcome in the general population) to investigate DU teratogenicity requires the designation of specific birth defects categories as the indicator of case status.

Rarity of outcome is diminished when more specific categories are aggregated into larger groupings; but if aggregation is done inappropriately, rather than aid in accurate assessment, it will add "noise" and diminish the extent to which the data can reflect a true association. Most malformations observed at birth originate in early embryonic development. Therefore, prenatal development processes would provide a meaningful basis for aggregation. Almost none of the available data regarding DU teratogenicity were gathered within such a framework. But studies that used the strategy of "lumping" data by organ system and those that gave detailed data for individual malformations could be "tweaked" for assessment from a developmental embryology perspective.

### Discussion of main epidemiological findings and suggestions of next steps

The Basra registry studies [43,44] are the starting point for discussion. The data are both profound and enigmatic. The very existence of the data is remarkable; the data are testimony to the commitment of a clinical research group – to their patients and to the potential of science to promote knowledge that can benefit generations to come.

The data are enigmatic for several reasons. There are jarring differences between the reported data and what would be expected based on malformation registries in the West. The most striking are:

i) The very low incidence of malformations reported pre-war. While the data indicate dramatic increases in incidence of malformations since the 1991 war, they start from such a low level in 1990, pre-war, that they often only reach the baseline Western levels by 1999–2000. Does the low Iraqi baseline reflect a truly lesser incidence of birth defects in Iraq at that time as compared to the West – possibly a reflection of the impact of the types of pollution and stress that are part and parcel of life in the "developed world"? Prior to the 1991 Gulf War, compared to the West, the Iraq environment was surely more pristine and the daily rhythms and challenges different. Or was detection of birth defects at the Basra study hospital less than complete? And, if so, was incomplete detection systematic or random?

ii) The malformation categories in which the data are presented. The categories do not correspond *in toto *to groupings used in the West. In the absence of detail explaining which abnormalities are included in which categories, inference becomes more difficult.

The hydrocephalus data are an example of the confusion that can result from the lack of clarity regarding which malformations were routinely documented and how they were grouped. It is implausible that there were no infants born with hydrocephalus at the Basra study hospital during the years 1991 – 1998 as that would mean no cases of hydrocephalus among about 100,000 births. It is hoped that this, and other unusual data features, e.g., no reported cases of microcephaly, 1990 – 2000, will eventually be clarified.

iii) The wholesale lumping together of infants identified as having multiple congenital malformations. From an analytic point of view information regarding the composition of the rapidly growing group is sorely missed. This category may be the "repository" for infants with hydrocephalus and microcephaly! Such malformations, and many others, often are not solo conditions.

iv) Also missing from the report is information on pre-and perinatal exposures of affected infants.

Aware of these imperfections, this substantial database remains an important source of information. In comparison to 1990 pre-war data, the data for subsequent years suggest that, in association with DU exposure, there are increased rates of:

Neural tube defects (NTDs),

Births with multiple congenital malformations,

Congenital heart diseases,

Cleft lip and palate,

The unusual skeletal malformation phocomelia

Congenital malformations in toto.

The **NTD**s reported in the Basra registry are anencephaly and meningomyelocele. Between 1990 and 1999–2000 the incidence of anencephaly rose from 2.5 to 17.4 per 10,000 births and of meningomyelocele from 7.0 to 11.3 per 10,000. The relative risk of these two NTDs among births in the study hospital for the years 1991–94, 1995–98, 1999–2000, in comparison to 1990, were 0.74, 1.31, and 2.91. By 1999–2000, the combined prevalence rate of anencephaly and meningomyelocele in the study hospital had reached 29/10,000. (If meningocele is not subsumed in another of the CNS categories, it is anomalous that there were no observed cases.)

(Anencephaly is a case in point regarding the difficulties relating time trends in frequency of occurrence of particular malformations in the Basra data with population data from the West. On the one hand, the incidence of anencephaly at birth has been declining in the West, 3.5/10,000 births in 1979–1980 vs. 2.3/10,000 in 1986–87 based on annual monitoring of hundreds of thousands of births by the U.S. Birth Defects Monitoring Program [67]. Between 1990 and 1998 the incidence of anencephaly among the Basra study newborns, rose from 2.5 in 1990 to 3.0 during the years 1991 – 94 and 3.6 in 1995 – 98.)

The Diwaniah registry study [46], designed specifically to assess prevalence of NTDs in a city that was bombarded with DU, found higher prevalence rates among births in 2000: 84/10,000 for anencephaly, meningomyelocele, menigocele, and encephalocele, 54/10,000 if the affected are limited to those diagnosed with anencephaly and meningomyelocele. Like the anencephaly rates inferred from the 1999 Guardian article [57], these rates are high in comparison to population rates reported in the West.

Without elaboration, the Diwaniah report mentions that data were gathered for each NTD case on a number of other potentially confounding factors and that those factors were not found to be relevant. With no detail, the rate of all NTDs among the 6124 births during an eight-month period a year previous (7/98–2/99) was also high, 54/10,000, though not quite as high as the 2000 rate. Neither the Basra nor the Diwaniah reports defined their malformation categories; it is unclear which Diwaniah data are more appropriate for comparison with Basra data.

With little detail, and adding the complexity of invoking male-mediated teratogenicity, the citizen-run ABDC case series flagged an excess of **Goldenhar Syndrome **(a clustering of malformations derivative from the 1^st ^and 2^nd ^brachial arches of the neural crest) among offspring of 1991 male Gulf War veterans [40]. (To re-iterate, only some of the Gulf War veterans were substantially exposed to DU.) A 1997 study by Araneta et al. [41] of two 30,000+ cohorts of veterans was conducted in response to the ABDC "flag". Given the rarity of the syndrome, that the observed 3-fold relative risk associating Goldenhar's with 1991 Gulf War service was not statistically significant, is less informative than the direction of the finding – consonant with other data. It is worth a note that renal agenesis/hypoplasia, an occasional component of Goldenhar Syndrome, was observed by Araneta et al, in other research [50], to be significantly more common among offspring born post-war to American 1991 Gulf War veterans than among their pre-war offspring.

The multiple underlying etiologies of **congenital hydrocephalus **include neural tube dysmorphology. Hydrocephalus as part of Goldenhar Syndrome is one such manifestation. Citizen activists did identify an excess of hydrocephalus among births downwind of the Socorro, New Mexico DU munitions testing site [38]. The 1999 Guardian article quoted from above also indicated an excess of hydrocephalus among Basra infants exposed *in utero *to DU aerosols [58]. That perplexing aspect of the Basra registry study – that those data indicate zero cases of hydrocephalus among the 100,000 births during the years 1990 – 98 and an unusually high incidence of the malformation, by any international standard, in 1999–2000 needs to be cited. As previously noted, in a personal communication a member of the Basra team asserted that the frequency of hydrocephalus has been elevated in Basra since 1991 [45].

**Multiple congenital malformations **is a broad category whose prevalence rose sharply in the data of the Basra registry studies, reaching 42/10,000 by 1999–2000. The relative risk of multiple congenital malformations (no further definition) for the years 1991–94, 1995–98, 1999– 2000 in comparison to 1990, are 1.13, 1.90, and 7.35. For various reasons it would be valuable to learn more about the malformation clusters being observed. For example, this category may be the unfortunately shadowed repository of infants born with congenital hydrocephalus in the years 1990 – 1998.

In the Basra study hospital the relative risk of **congenital heart diseases **for the years 1991–94, 1995–98, and 1999–2000 in comparison to 1990, was 2.4, 5.8, 8.3. In 1999–2000 the reported prevalence of congenital heart diseases was 14/10,000. By any Western standard even the 1999 – 2000 Basra rate is low. While many congenital heart disorders are not diagnosed immediately at birth and that is the only time point at which the Basra infants were assessed, U.S. data also limited to diagnosis at birth documented overall cardiovascular malformation rates upwards of 35/10,000 [67]. The breadth of cardiac malformations makes it particularly unfortunate that there is no detail about the types of defects recorded in Basra. The extent to which the Basra "congenital heart disease" conditions are congruent with the American "cardiovascular malformations" is not known.

Detail about the Basra cardiac cases would facilitate comparison with data from the Kuwaiti, 1986–1989 vs. 1992–2000, congenital heart defects study and the best of the US veterans studies. The post 1991 Gulf War rates of congenital heart defects overall and of numerous specific defects are high in the Kuwaiti study compared to data from the West. Abushaban et al. [56] found an overall post-war relative risk for congenital heart defects above 2 1/2 and statistically significant differences in incidence (pre vs. post) for 13 of 17 sub-categories. In the US veterans study of Araneta et al. two (of 14) types of congenital heart defects were identified as statistically more common among offspring of 1991 male Gulf War veterans than among offspring of other male veterans [50]. It is of note that certain cardiac malformations occur as part of Goldenhar's.

The Basra registry studies also show a dramatic rise in **cleft lip and palate**, from one case in a cohort of 12,000+ in 1990 to 21 cases in the 1999–2000 cohort of 26,000+. Compared to Western rates the1990 prevalence is remarkably low and even the 1999–2000 rate is low.

Unlike the categories cleft lip and palate and "congenital heart diseases", **phocomelia **is a specific and rare birth defect. Its changing prevalence in Basra since the 1991 war is a striking phenomenon. The prevalence of phocomelia rose explosively from 0 cases in 1990 and one in the four-year period 1991–94 to five in the following four years, and 32 in 1999–2000. As such, studies designed to document phocomelia in other contexts where DU munitions have been exploded could contribute significantly to causal inference. Another strategy might be to investigate the residential proximity of women delivering neonates with phocomelia to sites which generate DU-containing pollution. Previously a global rise in phocomelia incidence bespoke thalidomide teratogenesis.

Most striking about the elevated rates and numbers described above is that they continue to rise with time, dramatically so in 1999–2000. The same pattern is true through 2001 for total malformations; there have been annual increases since 1997. This increasing rate of occurrence of malformations could contribute to distinguishing causally related exposures from non-causal associations. The effects of most time-limited exposures diminish as time since exposure increases. With DU, refinement of causal pathways could demonstrate a mechanism that expresses burgeoning internal damage or chronicity and compounding of exposure. The categorical breakdown of the totality of birth defects observed among those born at the Basra study hospital in 2001 will provide further information about trends.

While particular malformations of several of the classes discussed above can be derivative from abnormal neural crest development, it is both premature and not our area of expertise to try to draw conclusions about the possibility that defects derive from common physiologic processes. However, there is a need to more carefully examine the issue; if the biology supports the notion that a single underlying mechanism is possible, it would support the plausibility of DU as a prime candidate. A trans-disciplinary study that provides careful histological comparison of birth defects produced in animals under controlled circumstances and those seen in epidemiological studies is also warranted.

In discussion of their findings through 1998, the Basra researchers stated that their data indicate an increase in birth defects in Basra beginning in 1995, four years after initial exposure. With the data through 2000 at hand, these writers' assessment is that it is difficult to distinguish between a gradual and ongoing increase in rates for various defects vs. a several year lag-to-onset of any elevation of rates. Specifying either of these distinctive temporal patterns for occurrence of birth defects could contribute to efforts to separate the teratogenic roles of DU and other possible antecedent causes, and also to efforts to discern the underlying mechanism that leads to teratogenesis. Both descriptions of the Basra pattern (a gradual rise, a lag-time-to-rise in frequency of occurrence of birth defects) are consonant with the Battelle Laboratory work that measured at minimum a 4-year dissolution and migration half-time for ceramic DU [5].

If it is only according to a multi-month timeframe that DU manifests its teratogenic potential, then the utility of the early post-1991 Gulf War American veterans studies is compromised. The 2003 study by Araneta et al. considered infants born only through 1993. It is because of its longevity that the Basra registry is providing substantial information.

It is noteworthy that the US veterans cohort study does not corroborate several other Basra findings regarding classes of malformations with elevated incidence (neural tube defects – anencephaly and meningomylocele, cleft lip and palate, births with multiple congenital malformations, phocomelia), likely because the designs of the studies were dissimilar. A forward extension of the study of Araneta et al. along with refinement of the exposure measure to allow for classification of Gulf War veterans by whether or not there was DU exposure in their particular theatre of combat and by characteristics of that exposure would be quite informative and is recommended. Inference from the reported statistical association between maternal 1991 Gulf War deployment and hypospadias and epispadias, as a currently completely uncorroborated finding, would also benefit from a refined and expanded analysis.

The issue of how to distinguish the role of DU from that of other suspected teratogens is serious and complex. The response to this challenge is built on the interface of laboratory research and population studies; its glue is the application of epidemiological principles of inference. Laboratory and animal research are proceeding apace and are suggesting plausible pathways by which internalized DU aerosols could be mutagenic and/or teratogenic. As animal studies come to provide more detail about the internal migration of inhaled ceramic DU and its decay particles, inference regarding possible teratogenic pathways for specific birth defects can be refined.

A 1994 U.S. General Accounting Office report identified 21 reproductive toxicants and teratogens, including DU, that were present in the 1991 Gulf War environment [68]. A commonality of excessive occurrence of a particular birth defect among offspring of American veterans, and offspring of Iraqi veterans and resident civilians would decrease the likelihood that certain of those 21 toxins had a causal role in the elevated rate of occurrence of that defect among American veterans' offspring. For example, Iraqis did not receive the "medications and vaccines administered to Gulf War veterans". Therefore, a similar or identical excess of a particular class of birth defects among offspring of DU-exposed Americans and Iraqis could not uniformly be attributed to those medications and vaccines.

It is from this vantage that the Socorro case study is of particular significance. By trans-national standards, the rate of occurrence of hydrocephalus in Basra during the years 1999 and 2000 was very high. (This, notwithstanding the need for clarification of the 1990–1998 registry data regarding occurrence.) If DU is the sole, or one of a small group of, risky exposure(s) shared by residents of the Iraqi war region and residents of the rural Socorro, New Mexico munitions testing region, then the likelihood of a causal role for DU in the genesis of hydrocephalus is increased.

More generally, serious effort needs to be directed toward disentangling the role of DU from that of other potential teratogens in tandem with which DU exposure has frequently occurred. This task becomes less daunting, though more urgent, as the contexts in which DU munitions have been exploded increases. The identities of the "other potential teratogens" disbursed into the environment by the crash of an airplane carrying DU in a civilian area differ, at least somewhat, from those disbursed by DU fires in a combat zone. In response to "widespread distress" about crash-associated risk, a theoretical physics-based model of the 1992 event was developed. While that theoretical study did not include any assessment of the health status of the exposed population (and their offspring), the authors concluded that it was "improbable" that the DU that had burnt and aerosolized as a result of the crash precipitated health problems [69]. Such a purely theoretical approach seems inadequate, especially in light of the popular perception of a post-crash regional increase in malformed births [61]. Furthermore, associations documented in an unexpected context that cohere with findings of planned analyses are highly informative. Conversely, absence of observed associations in small, unexpectedly exposed populations would be less informative.

In addition to Socorro, New Mexico there are 50 other US sites where DU munitions are/have been developed, produced, tested. How many of these sites are located in areas where comprehensive birth defects registries exist? What about other countries? Could assays for DU biomarkers be done on groups of male and female parents of children with and without birth defects resident near such facilities?

For the Iraqi population the 1991 Gulf War was the prelude to various new exposures and circumstances that could be teratogenic – sanctions-induced deprivations such as poverty, malnutrition and degradation of the health care infrastructure. But such circumstances, without specific chemical or radiologic exposures, do not lead to the observed pattern of increasing rates for classes of congenital malformations, notwithstanding the fact that malnutrition does contribute to certain birth defects. If a comparable birth defects registry (1990–2000) were available for births in a section of northern Iraq not exposed to DU bombardment, it could help distinguish between war-induced and post-war exposures.

A cohort study from Kerala, India is a particularly apropos example of a well-executed investigation that was able to detect differences in the occurrence of birth defects (and other untoward pregnancy outcomes) among population groups [70]. In a genetic epidemiological and fertility survey conducted among 700,000 people in regions with normal background radiation (85 to 110 mR/yr) and high background radiation (735 – 563 mR/yr) – from thorium monazite in the soil – Padmanabham et al used personalized, direct contact with families to document a statistically significant increase in congenital malformations and other birth outcomes in the area with higher background exposure. Besides ionizing radiation, consanguinity and nearness of spouse's birthplace were included as additional risk factors for each birth outcome. This study is a model for an investigation of the incidence of birth defects (and other pregnancy outcomes) in regions of Iraq with and without contamination by DU aerosols. Ideally the regions being compared would be as similar as possible on other criteria including distribution of occupations and religion, economic situation, culture, or would allow for "control" of differences, as in the model of the Kerala study. (Of course, this study is also informative because though the radiation exposure in the Kerala region is due to radon, the case for teratogenicity related to increased radiation exposure is made.)

The study of Abushaban et al [56] is an assessment of the impact of DU on one class of birth defects in the absence of sanctions. In Kuwait, where there was DU (and other wartime) exposure(s) but no post-war sanctions, the post-war incidence of cardiac malformations overall and of numerous specific sub-categories was elevated. Kuwaiti trend data regarding prevalence of other classes of birth defects, particularly those elevated in Iraqi and other DU-exposed databases, could be highly informative.

It would be poor science to not acknowledge another potent resource for investigating human DU teratogenicity. Even as shoe leather epidemiology generated the Socorro Case Study, the establishment of opportunities for interaction between scientists and activists might reveal other suspect clusters of birth defects. There are citizen activist groups organized around more than one of the U.S. DU munitions testing sites. Are activists aware of facts-on-the-ground that should inform scientific studies?

From the grassroots to the international context: Resolution of the longstanding jurisdictional conflict regarding representation of the U.N. position on potential health effects of radiation exposure is needed. In 1959 the IAEA and the WHO signed an agreement (WHA 12.40) that has been used by the IAEA to constrain WHO's health-related radiation research and it may also be affecting the UN approach to DU research [71].

There is a serious need for careful epidemiological research that can elucidate the relationship between DU exposure and specific classes of birth defects. High quality, well-funded registries to monitor prevalence of individual classes of birth defects must be the norm in countries where DU munitions have been exploded, in the home countries of veterans who have gone abroad and waged war using DU munitions and in the countries where DU munitions are manufactured. As well, there is a critical need for epidemiological investigations that incorporate companion bioassays to assess internal parental exposure to DU (and other suspected teratogens).

While this article focuses exclusively on congenital malformations there is documentation that in other contexts very early miscarriage is another reproductive endpoint affected by radiation exposure. This implies a radiation dose-response effect such that there is a ceiling in the proportionality between radiation exposure and frequency of congenital malformations. The observed drop in the birth rate in many European countries 7 – 9 months after the Chernobyl disaster presumably relates to an excess of miscarriages induced by that event – obscuring the underlying teratogenic impact. The Basra data report (Table 1) is consistent with a drop in number of births at the study hospital 7 – 9 months subsequent to the January 1991 regional use of DU weaponry. But whether the lower number of births in 1991 is due to increased miscarriage of damaged embryos, to reduced conceptions because of the more general vicissitudes of war or to some other factor cannot be determined with the available information.

There are three broad categories into which epidemiological assessment of data regarding a causal association between a potential risk factor and an outcome distribute: the existence of association (be it positive or negative), lack of association, the conclusion that the extant data are inadequate for inference. Data are never perfect, hence it is incumbent on the epidemiological/public health analyst to distinguish between situations where the data are so imperfect that no valid inference can be drawn and those where valid scientific assessment allows for attribution of risk. Regarding the teratogenicity of parental prenatal exposure to DU aerosols, the evidence, albeit imperfect, indicates a high probability of substantial risk. Good science indicates that depleted uranium weapons should not be manufactured or exploded.

## List of abbreviations

AFFRI: Armed Forces Radiobiology Research Institute (given in the text)

DU: depleted uranium

IAEA: International Atomic Energy Agency

no.: number

NTD: neural tube defect

vs.: versus

WHO: World Health Organization

## Competing interests

The author(s) declare that they have no competing interests.

## Authors' contributions

RH led the identification of and analysis of the epidemiological studies and took the lead on writing the bulk of the manuscript. DB took the lead on reviewing the toxicological data. He also reviewed the entire manuscript many times and participated in discussions and editing of the entire manuscript. BP researched specific points of detail for the manuscript, participated in discussions and editing, reviewed the entire manuscript and contributed to writing some sections.
